# Identification and Functional Analysis of Targets of Dehydrodiisoeugenol in Bladder Cancer Based on Chemoproteomics-Based Profiling

**DOI:** 10.3390/ph19040651

**Published:** 2026-04-21

**Authors:** Zhao Zhai, Fan Wu, Guoli Sheng, Bin Jia, Bolin Jia, Peng Du, Yong Zhang

**Affiliations:** 1 Key Laboratory of Carcinogenesis and Translational Research (Ministry of Education/Beijing), Department of Urology, Peking University Cancer Hospital & Institute, Beijing 100089, China; 2 Department of Urology, National Cancer Center/National Clinical Research Center for Cancer/Cancer Hospital, Chinese Academy of Medical Sciences and Peking Union Medical College, Beijing 100021, China

**Keywords:** dehydrodiisoeugenol (DHE), ABPP, bladder cancer, PTPN1

## Abstract

**Background/Objectives:** The clinical management of bladder cancer is severely impeded by high recurrence rates and the rapid emergence of chemoresistance, necessitating the discovery of novel therapeutic agents with distinct mechanisms of action. Dehydrodiisoeugenol (DHE), a bioactive neolignan, exhibits potent anti-tumor efficacy, yet its direct molecular targets and mode of action remain elusive. **Methods**: To deconvolute the mechanism of DHE, we integrated a phenotypic screening approach using 2D cell lines and 3D patient-derived organoids with a chemoproteomics-based activity-based protein profiling (ABPP) strategy. We synthesized a functionalized photoaffinity probe to capture the specific interactome of DHE under physiological conditions and validated targets via cellular thermal shift assays (CETSA), quantitative mass spectrometry, and 100 ns molecular dynamics (MD) simulations. **Results**: DHE exhibited potent dose-dependent cytotoxicity in bladder cancer cells, with IC50 values of 39.23 μM in T24 and 34.58 μM in 5637 cells. In 3D patient-derived organoids, DHE significantly reduced viability (*p* < 0.0001). Using a dual-filtering ABPP strategy, we identified 65 high-confidence candidate targets, prioritizing PTPN1 (PTP1B) as the primary functional interactor. Comparative molecular docking and 100 ns MD analyses showed that multiple stereoisomers of DHE could adopt plausible PTPN1-binding modes. Mechanistically, organoid proteomics indicated that DHE engagement with PTPN1 disrupts ER membrane homeostasis, thereby modulating the PI3K-Akt signaling axes. **Conclusions**: These findings establish PTPN1 as a critical druggable vulnerability in bladder cancer and define the molecular basis for the therapeutic potential of DHE. This study highlights the power of combining chemoproteomics with physiological 3D models to accelerate the translation of natural products into precision cancer therapies.

## 1. Introduction

Bladder cancer is the 10th most commonly diagnosed cancer worldwide, with approximately 614,000 new cases and 220,000 deaths reported annually [1]. It remains one of the most prevalent and burdensome malignancies of the urinary system, exhibiting a striking gender disparity where incidence rates are nearly four times higher in men than in women [2]. In China, the clinical burden is particularly acute; incidence and mortality rates have shown a steady upward trend over the past decade, largely attributed to an aging population and high smoking prevalence [3,4]. Despite advancements in surgical resection and chemotherapy, the disease is characterized by a formidable rate of recurrence and progression, reaching up to 70% within five years for non-muscle-invasive cases, which necessitates the identification of novel therapeutic agents [5,6,7]. Despite advancements in surgical resection, chemotherapy, and emerging immunotherapies, the prognosis for patients with advanced or metastatic disease remains suboptimal due to the rapid development of chemoresistance [8,9]. The molecular heterogeneity of bladder cancer necessitates the identification of novel therapeutic agents that operate through distinct mechanisms to circumvent existing resistance pathways and improve long-term clinical outcomes [10].

Natural products, with their vast structural diversity and refined bioactivity, offer an unparalleled reservoir for drug discovery [11,12]. Dehydrodiisoeugenol (DHE), a prominent neolignan derived from Myristica fragrans, has recently emerged as a compelling candidate due to its pleiotropic anti-tumor properties. Preliminary evidence suggests that DHE effectively suppresses the malignant hallmarks of bladder cancer, including cell proliferation, colony formation, and lateral migration in various in vitro models [13,14,15,16]. Furthermore, our initial evaluations in 3D patient-derived organoid models—which more faithfully recapitulate the tumor microenvironment—confirm that DHE compromises tumor viability and structural integrity.

However, a significant bottleneck in the clinical translation of DHE is the lack of a defined molecular target. While phenotypic screens have established its efficacy, the direct interactome through which DHE exerts its biological effects remains largely uncharacterized. Traditional proteomic approaches often fail to capture the transient or specific interactions between small molecules and their protein receptors in the complex cellular milieu [17,18]. This knowledge gap not only obscures the precise mechanism of action but also hinders the rational design of more potent derivatives, emphasizing the urgent need for a systematic, unbiased target identification strategy.

In this study, we employed a chemical proteomics-based strategy, specifically activity-based protein profiling (ABPP) [19,20], to map the direct targets of DHE in bladder cancer. By developing a DHE-derived photoaffinity probe, we successfully captured the drug’s interactome under physiological conditions. Integrated analysis of quantitative mass spectrometry and organoid proteomics identified PTPN1 (Protein Tyrosine Phosphatase, Non-Receptor Type 1), a key phosphatase localized to the endoplasmic reticulum (ER) membrane, as the primary functional target [21,22,23]. Our findings not only elucidate a novel mechanism of DHE-mediated tumor suppression but also highlight PTPN1 as a druggable vulnerability in bladder cancer therapy [17].

## 2. Results

### 2.1. DHE Inhibits the Proliferation, Colony Formation, and Migration of Bladder Cancer Cells In Vitro

**Figure 1:**
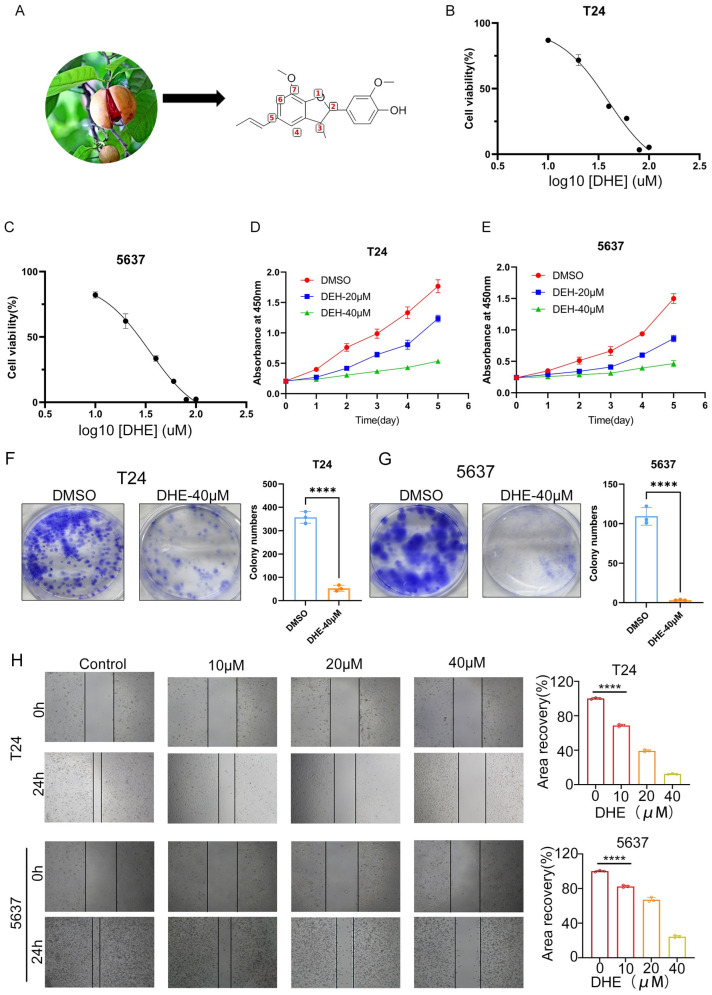
DHE suppresses the proliferation and migration of T24 and 5637 bladder cancer cells. (**A**) The natural source (Myristica fragrans) and chemical structure of DHE. Atom numbering of the 2,3-dihydro-1-benzofuran core is shown for clarity; the stereogenic centers are located at C-2 and C-3. (**B**,**C**) Dose-response curves of T24 and 5637 cells treated with indicated concentrations of DHE for 48 h, as measured using the CCK-8 assay. (**D**,**E**) Proliferation curves of T24 and 5637 cells treated with DMSO or DHE (20, 40 μM) over 5 consecutive days. (**F**,**G**) Representative images and statistical quantification of colony formation assays for T24 (**F**) and 5637 (**G**) cells following DHE treatment. (**H**) Wound healing assays evaluating the migratory ability of T24 and 5637 cells treated with increasing concentrations of DHE (0, 10, 20, 40 μM). Representative micrographs at 0 h and 24 h are shown on the left; quantitative analysis of the area recovery percentage is shown on the right. Data are presented as Mean ± SD (*n* = 3). **** *p* < 0.0001 vs. DMSO group (*t*-test or one-way ANOVA).

To explore novel therapeutic options for bladder cancer, we investigated the bioactivity of Dehydrodiisoeugenol (DHE), a natural lignan derived from Myristica fragrans (Figure 1A). We utilized two human bladder cancer cell lines, T24 and 5637, to evaluate the inhibitory effects of DHE on malignant phenotypes. Initially, CCK-8 assays were performed to assess the cytotoxicity of DHE. The IC50 values for T24 and 5637 cells were 39.23 μM and 34.58 μM, respectively. As shown in Figure 1B,C, DHE treatment for 48 h significantly reduced the viability of both T24 and 5637 cells in a dose-dependent manner.

To further characterize its impact on cell growth kinetics, we monitored cell proliferation over a 5-day period. Consistent with the viability data, DHE at concentrations of 20 μM and 40 μM markedly suppressed the growth rates of both cell lines compared to the DMSO-treated control group (Figure 1D,E). We next performed colony formation assays to evaluate the effect of DHE on the independent survival and reproductive integrity of single cancer cells. Treatment with 40 μM DHE led to a dramatic reduction in both the number and size of colonies in T24 and 5637 cells (Figure 1F,G, *p* < 0.01), suggesting that DHE effectively blocks the long-term proliferative potential and population expansion of bladder cancer cells.

Given that migration is a hallmark of cancer progression and metastasis, we employed a wound-healing assay to examine the effect of DHE on lateral cell motility. While the control group exhibited robust wound closure within 24 h, DHE treatment (10, 20, and 40 μM) significantly hindered the migration of T24 and 5637 cells into the denuded area (Figure 1H). Quantitative analysis of the area recovery percentage confirmed that DHE inhibits bladder cancer cell migration in a dose-dependent fashion, highlighting its potential to suppress tumor invasiveness.

### 2.2. DHE Exhibits Potent Anti-Tumor Activity in Physiologically Relevant 3D Organoid Models

**Figure 2:**
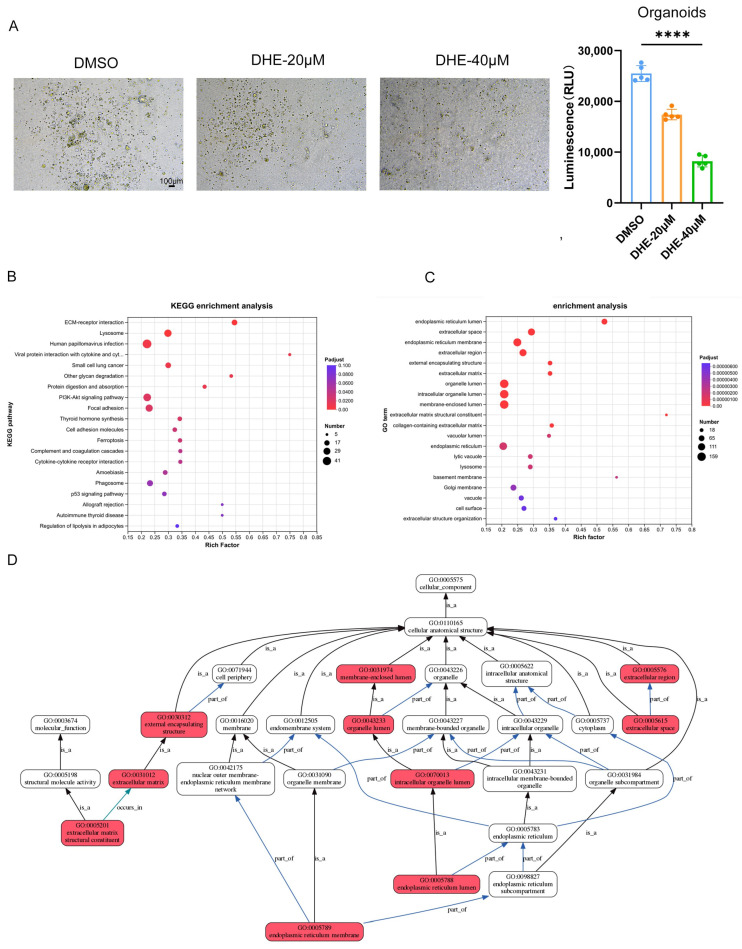
DHE suppresses the proliferation of bladder cancer 3D organoid models. (**A**) Representative bright-field micrographs (left) and luminescence-based viability quantification (right) of bladder cancer organoids following 72 h of treatment with DMSO or DHE (20, 40 μM). Scale bar: 100 μm. Data are presented as Mean ± SD (*n* = 3). (**B**) KEGG pathway enrichment analysis of differentially expressed proteins (DEPs) in DHE-treated organoids. The size of the dots represents the number of proteins, and the color represents the adjusted *p*-value. (**C**) GO enrichment analysis categorizing DEPs into biological processes (BP), cellular components (CC), and molecular functions (MF). (**D**) Directed Acyclic Graph (DAG) illustrating the hierarchical relationships of enriched GO terms. Red boxes highlight significant enrichment in the endoplasmic reticulum membrane and membrane-enclosed lumen, pointing toward a collapse of membrane-associated homeostasis. Differentially expressed proteins (DEPs) were identified using a student’s *t*-test with thresholds of *p* < 0.05 and a fold change (FC) >1.2 or <0.83. The False Discovery Rate (FDR) was controlled at ≤1%. Data are presented as Mean ± SD (*n* = 3). **** *p* < 0.0001 vs. DMSO group (*t*-test or one-way ANOVA).

To bridge the gap between in vitro cell line studies and clinical relevance, we established a 3D bladder cancer organoid model that better recapitulates the structural and functional complexity of the tumor microenvironment. Consistent with our 2D findings, treatment with DHE led to a striking, dose-dependent reduction in organoid size and density (Figure 2A). Quantitative luminescence-based viability assays confirmed that DHE at 20 μM and 40 μM significantly decreased the Relative Light Units (RLU) compared to the DMSO control group (*p* < 0.0001), demonstrating that DHE effectively compromises the survival and expansion of bladder cancer cells within a 3D architecture.

To systematically investigate the molecular mechanisms underlying DHE-induced growth inhibition, we performed quantitative proteomic analysis on the treated organoids. KEGG pathway enrichment analysis of the differentially expressed proteins (DEPs) revealed a significant impact on several hallmark pathways of cancer cell death and survival, most notably ECM-receptor interaction, the p53 signaling pathway, and the PI3K-Akt signaling pathway (Figure 2B). To further define the cellular response, we performed Gene Ontology (GO) enrichment analysis. GO enrichment analysis revealed that DEPs were significantly enriched in terms associated with the endoplasmic reticulum (ER) membrane, the extracellular matrix, and membrane-enclosed lumens (Figure 2C). A Directed Acyclic Graph (DAG) analysis of these GO terms further visualized a highly connected cluster of perturbations centered on ER homeostasis and organelle-lumen integrity (Figure 2D).

### 2.3. Development and Biological Validation of a DHE-Derived Photoaffinity Probe for Target Identification

**Figure 3:**
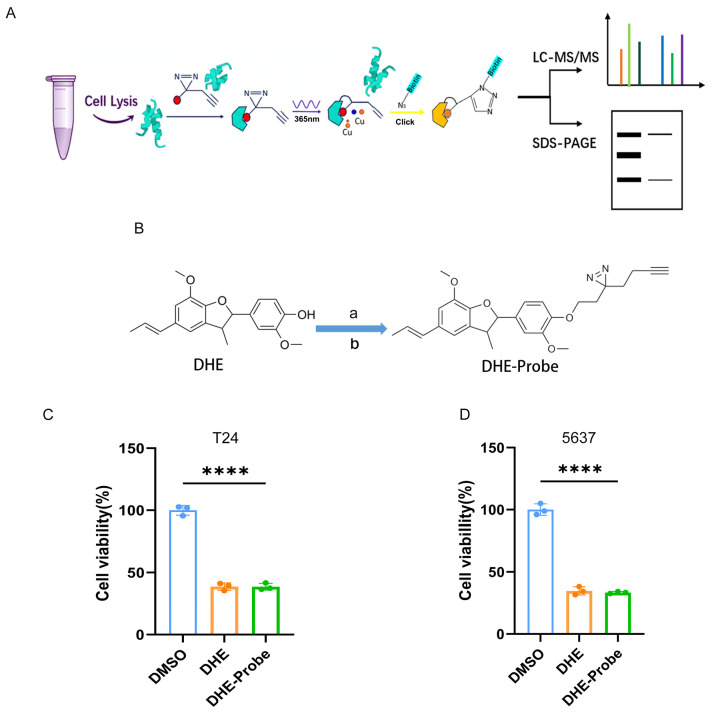
Synthesis and biological validation of a DHE-derived photoaffinity probe. (**A**) Schematic representation of the ABPP (Activity-Based Protein Profiling) workflow. The process includes cell lysis, probe incubation, UV-induced cross-linking, click chemistry-mediated biotinylation, and streptavidin-based enrichment followed by LC-MS/MS or SDS-PAGE analysis. (**B**) Synthetic route of the DHE-Probe. Reaction conditions: (a) diazirine–alkyne linker, (b) K_2_CO_3_, DMF, 65 °C, 16 h. The final probe includes a photo-cross-linker and an alkyne handle for target capturing. (**C**,**D**) Comparison of the anti-proliferative effects of DMSO, DHE, and DHE-Probe (40 μM) in T24 (**C**) and 5637 (**D**) bladder cancer cells. Cell viability was measured 24 h post-treatment. Data are presented as Mean ± SD (*n* = 3). **** *p* < 0.0001 vs. DMSO group (*t*-test or one-way ANOVA).

Having established the potent anti-tumor activity of DHE and its association with ER- and membrane-related pathways, we next sought to identify its direct molecular targets using a chemical proteomics approach. To this end, we designed and synthesized a DHE-derived photoaffinity probe (DHE-Probe) by incorporating a diazirine group for UV-induced covalent cross-linking and an alkyne handle for subsequent click chemistry-mediated labeling and enrichment (Figure 3B).

Based on this probe, we established an activity-based protein profiling (ABPP) workflow to capture DHE-binding proteins (Figure 3A). In this strategy, cell lysates were incubated with the DHE-Probe, followed by irradiation with 365 nm UV light to induce covalent cross-linking between the diazirine moiety of the probe and interacting proteins. Subsequently, a biotin-azide tag was conjugated via copper-catalyzed click chemistry, allowing selective enrichment of labeled proteins using streptavidin beads. The enriched proteins were then visualized via SDS-PAGE or subjected to LC-MS/MS for target identification (Figure 3A).

A critical prerequisite for successful target identification is that the chemical modification of the parent compound should not substantially impair its biological activity. We therefore compared the cytotoxic effects of the DHE-Probe and the parent compound DHE in T24 and 5637 bladder cancer cells. Cell viability assays showed that the DHE-Probe retained an inhibitory effect nearly equivalent to that of DHE in both cell lines (Figure 3C,D), indicating that the introduction of the photoaffinity group and alkyne handle did not markedly alter the pharmacological properties of the parent compound. These results support the suitability of the DHE-Probe for subsequent target identification studies.

### 2.4. The DHE-Probe Exhibits Robust and Dose-Dependent Labeling of the Bladder Cancer Proteome

**Figure 4:**
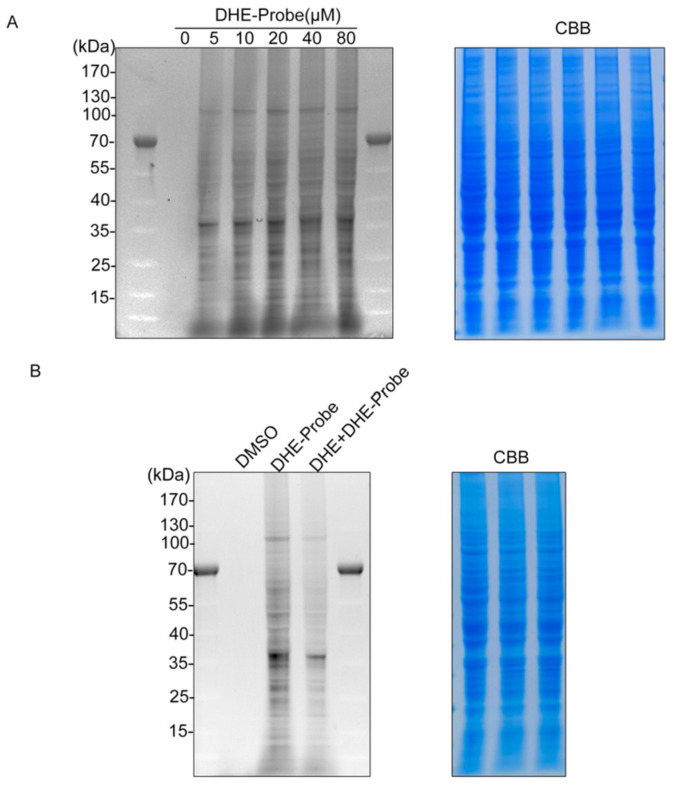
Gel-based ABPP validates specific protein labeling by the DHE-Probe. (**A**) Concentration-dependent labeling of the T24 cell proteome. Cell lysates were incubated with indicated concentrations of DHE-Probe (0, 5, 10, 20, 40, 80 μM), followed by UV cross-linking (365 nm) and click chemistry. The left panel shows the labeling profile (fluorescence/chemiluminescence); the right panel shows Coomassie Brilliant Blue (CBB) staining as a loading control. (**B**) Competition assay for binding specificity. Lysates were either treated with the DHE-Probe alone or pre-incubated with excess unlabeled DHE (Competition) prior to probe labeling. The significant reduction in band intensity in the competition lane (middle) confirms the specificity of the interaction. CBB staining (right) indicates equal loading. Molecular weight markers (kDa) are indicated on the left of each gel. Representative images from three independent experiments are shown.

After confirming that the DHE-Probe retains biological activity, we utilized it to visualize the landscape of DHE-binding proteins in T24 cell lysates. Following incubation with increasing concentrations of the DHE-Probe (0–80 μM) and subsequent UV-induced cross-linking, the proteome was subjected to click chemistry with a fluorescent/biotin tag. Gel-based profiling revealed that the DHE-Probe labeled a discrete set of proteins in a clear dose-dependent manner (Figure 4A). Coomassie Brilliant Blue (CBB) staining confirmed equal protein loading across all lanes, ensuring that the increase in signal was due to specific probe binding rather than protein concentration variations.

Competition assays confirm the specificity of the DHE-protein interaction. To distinguish specific binding from non-specific background labeling, we performed a competitive displacement assay. Cell lysates were pre-incubated with an excess of unlabeled parent compound (DHE, 100 μM) before the addition of the DHE-Probe (20 μM). As shown in Figure 4B, the signal intensity of the major labeled bands was significantly attenuated in the presence of excess DHE. This successful competition demonstrates that the DHE-Probe and the parent drug compete for the same binding pockets, confirming that the captured proteins are genuine, specific targets of the DHE scaffold. These results provide the foundational physical evidence required to proceed with large-scale proteomic identification of the DHE-interactome.

### 2.5. Unbiased Screening Identifies PTPN1 as a High-Confidence DHE Interactor

**Figure 5:**
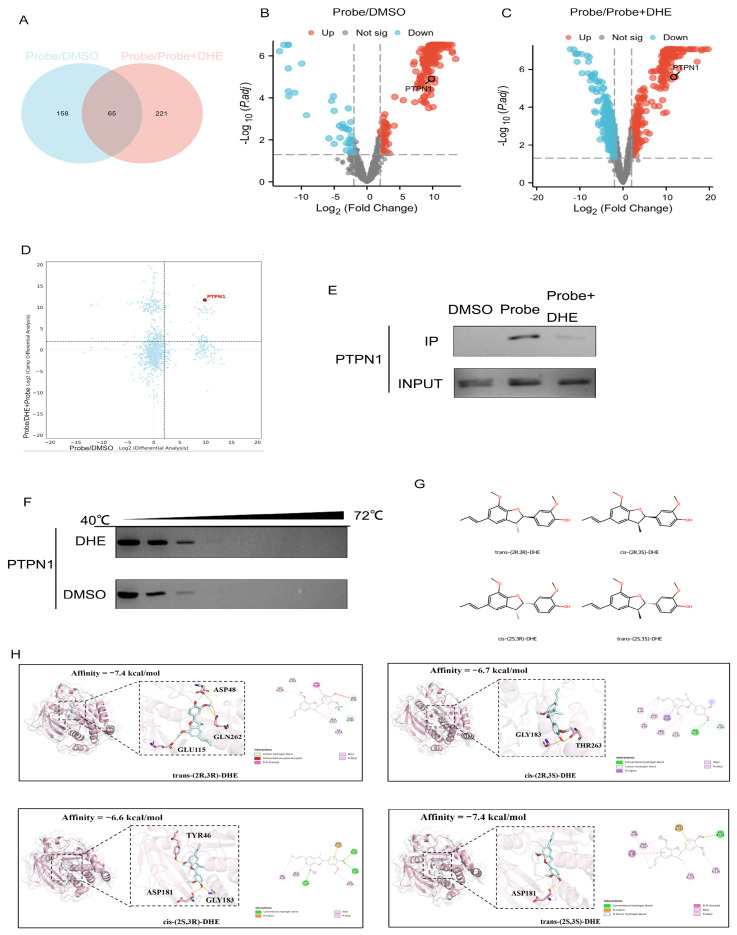
Identification and biophysical validation of PTPN1 as the direct target of DHE. (**A**) Venn diagram showing the overlap of 65 potential target proteins identified through enrichment (DHE-Probe/DMSO, logFC > 2) and competition (Probe/DHE + Probe, logFC > 2) LC-MS/MS datasets. (**B**,**C**) Volcano plots displaying the distribution of proteins in the enrichment (**B**) and competition (**C**) groups. PTPN1 is highlighted as a significantly enriched and competed target. (**D**) Scatter plot integrating the differential analysis of enrichment vs. competition, positioning PTPN1 as a top-tier candidate target. (**E**) Western blot analysis of PTPN1 following an ABPP pull-down assay. The Probe captures PTPN1, and the signal is attenuated by competition with excess unlabeled DHE. Input serves as a loading control. (**F**) CETSA results showing the thermal stabilization of PTPN1 by DHE across a temperature range (40–72 °C). DHE treatment protects PTPN1 from thermal denaturation compared to DMSO. (**G**,**H**) Molecular docking analysis of DHE with PTPN1. (**G**) The four stereoisomers of DHE. (**H**) Comparative docking affinities of the four stereoisomers of DHE toward PTPN1. LC-MS/MS analyses were performed using three biological replicates per group, and representative immunoblots from three independent experiments are shown in panels (**E**,**F**).

To definitively identify the molecular targets of DHE, we performed a comprehensive LC-MS/MS analysis using the ABPP workflow established in Section 2.3 and illustrated in Figure 3. We employed a dual-filtering strategy: enrichment (DHE-Probe vs. DMSO, logFC > 2) and competition (DHE-Probe vs. DHE + Probe, logFC > 2). The intersection of these datasets yielded 65 candidate proteins that were both significantly enriched by the probe and effectively competed by the parent drug (Figure 5A–C). By integrating these candidates with our previous organoid proteomic data—which highlighted endoplasmic reticulum (ER) membrane disruption and PI3K-Akt signaling (Figure 2)—we prioritized PTPN1 (also known as PTP1B) as the primary candidate (Figure 5D). The rationale for prioritizing PTPN1 over other candidates was twofold: first, it exhibited the highest consistency across enrichment and competition datasets; second, its spatial localization to the ER-cytosol interface aligns with the proteomic signature of organoid membrane disruption. PTPN1 is a key phosphatase localized to the ER membrane, making it a perfect functional and spatial match for the observed DHE-induced phenotype.

To verify the MS-based identification, we performed an affinity pull-down followed by Western blotting. The DHE-Probe successfully captured endogenous PTPN1 from T24 cell lysates, whereas the addition of excess unlabeled DHE significantly abolished this capture, confirming a specific and competitive binding interaction (Figure 5E). To further demonstrate direct binding in a cellular environment, we conducted a Cellular Thermal Shift Assay (CETSA). DHE treatment significantly increased the thermal stability of PTPN1 compared to the DMSO control across a temperature gradient (40 to 72 °C), providing strong evidence of a direct drug-protein physical interaction (Figure 5F).

To address the stereochemical discrepancy identified during revision, we re-performed the computational analysis using all four E stereoisomers of DHE, namely trans-(2R,3R)-DHE, cis-(2R,3S)-DHE, cis-(2S,3R)-DHE, and trans-(2S,3S)-DHE. Comparative docking showed that trans-(2R,3R)-DHE and trans-(2S,3S)-DHE yielded the most favorable docking affinities (both −7.4 kcal/mol), whereas cis-(2R,3S)-DHE and cis-(2S,3R)-DHE showed less favorable scores (−6.7 and −6.6 kcal/mol, respectively). Taken together, these results indicate that multiple E stereoisomers of DHE can adopt plausible binding modes within the PTPN1 pocket. The four stereoisomers and their comparative docking affinities are shown in Figure 5G,H.

### 2.6. Comparative MD Analysis Supports the Binding of DHE Stereoisomers to PTPN1

**Figure 6:**
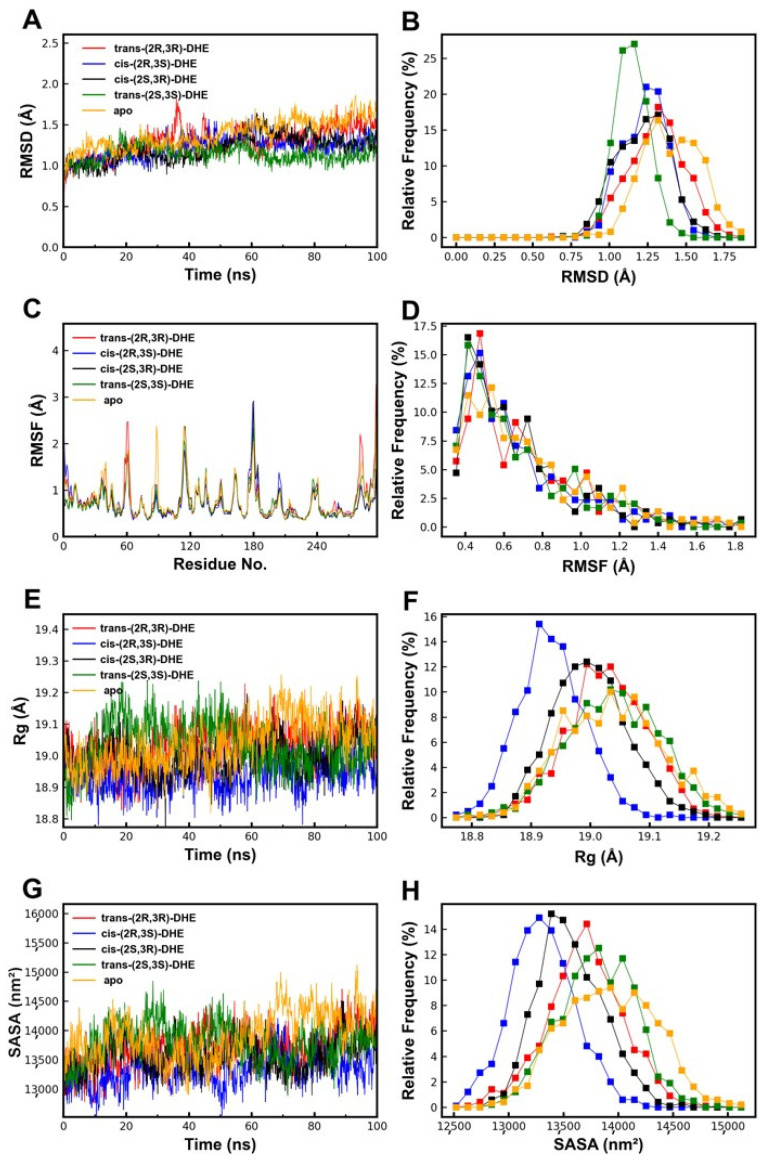
Comparative molecular dynamics (MD) analysis of the four stereoisomer–PTPN1 complexes. (**A**,**B**) RMSD trajectory. (**C**,**D**) RMSF profile. (**E**,**F**) Radius of gyration (Rg). (**G**,**H**) Solvent accessible surface area (SASA).

To evaluate the dynamic stability of the interaction between DHE and its target, PTPN1, under simulated physiological conditions, we performed a 100 ns molecular dynamics (MD) simulation on four stereoisomer-PTPN1 complexes. Overall, all four stereoisomers were able to adopt plausible binding modes within the PTPN1 pocket, although they showed differences in trajectory behavior and conformational sampling. Comparative RMSD, RMSF, Rg and SASA indicated that ligand binding was structurally feasible across multiple stereoisomers (Figure 6A-H). These findings support the structural plausibility of DHE binding to PTPN1.

In addition, we conducted analyses of intermolecular hydrogen bonds (HBonds) and the free energy landscape (FEL). The HBonds analysis revealed frequent interactions, with the number of HBonds across the four isomers fluctuating between 0 and 2 (Figure S1, in the Supplementary Materials). These hydrogen bonds—characterized by both their dynamic nature and their persistence—play a pivotal role in anchoring DHE within the binding pocket of the PTPN1 protein. The FEL maps revealed distinct conformational basins for the stereoisomer–PTPN1 complexes, supporting the structural feasibility of DHE binding to PTPN1 across multiple stereoisomers (Figure S2, in the Supplementary Materials).

## 3. Discussion

The clinical management of bladder cancer remains constrained by high recurrence rates and the eventual emergence of chemoresistance to standard-of-care regimens [24,25]. In this study, we established a chemoproteomics-driven framework that prioritizes PTPN1 as a high-confidence direct interactor of dehydrodiisoeugenol (DHE), a natural neolignan that suppresses bladder cancer progression in both 2D cell models and physiologically relevant 3D organoids. By bridging the gap between ethnopharmacology and precision chemical biology, our findings reveal a novel mechanism of action centered on the disruption of endoplasmic reticulum (ER) membrane homeostasis and the modulation of oncogenic signaling.

The identification of PTPN1 (PTP1B) as the primary target of DHE is particularly significant given its strategic localization on the cytoplasmic face of the ER membrane [26]. Our quantitative proteomic analysis of DHE-treated organoids highlighted a striking enrichment of proteins associated with the ER membrane and membrane-enclosed lumens. PTPN1 is a master regulator of receptor tyrosine kinase (RTK) signaling and lipid metabolism; its inhibition has been previously linked to the induction of ER stress and hypersensitivity to oxidative cell death [27,28,29]. The convergence of our ABPP-based target discovery and unbiased organoid proteomics strongly suggests that DHE induces a “proteostatic crisis” at the ER-cytosol interface in bladder cancer cells.

From a structural perspective, the biophysical and computational results support the feasibility of DHE binding to PTPN1. The DHE-Probe effectively captured endogenous PTPN1 in a competitive manner, while CETSA results confirmed a clear thermal stabilization of the protein upon drug binding. Comparative molecular dynamics (MD) simulations further showed that multiple stereoisomers of DHE could adopt plausible PTPN1-binding modes. While our biophysical data confirm direct binding, the induction of ER stress and PI3K-Akt modulation likely stems from the DHE-mediated inhibition of PTPN1’s phosphatase activity. Future studies employing CRISPR-mediated knockdown or enzymatic activity assays will be essential to definitively characterize PTPN1 as the primary mechanistic driver. While our current study provides a robust snapshot of the proteomic remodeling associated with DHE-mediated tumor suppression, we recognize that the evaluation of early signaling events (within 1–6 h) and dose-dependent global proteomic shifts would further strengthen the mechanistic resolution of the PTPN1-ER stress axis. Future studies utilizing time-resolved phospho-proteomics will be essential to map the immediate downstream consequences of DHE-PTPN1 engagement before the onset of homeostatic collapse. Moreover, although PTPN1 was preferentially identified as the primary target, given that natural neolignans (such as DHE) typically possess multi-target pharmacological activities, these off-target interactions may collectively contribute to the observed antitumor efficacy. Elucidating the specific functional contributions of these secondary targets remains a key objective for future research. Notably, the utilization of patient-derived organoids in this study significantly enhances the translational value of our findings. Unlike traditional 2D cultures, these 3D models recapitulate the structural and functional complexity of the tumor, providing a more rigorous test for drug efficacy [30,31]. The dose-dependent reduction in organoid viability and the subsequent proteomic remodeling confirm that the DHE-PTPN1 axis is a viable therapeutic vulnerability in a setting that mimics the clinical tumor microenvironment.

In conclusion, our study establishes DHE as a potent, PTPN1-targeting lead compound for bladder cancer therapy. By integrating ABPP-based chemical proteomics with 3D organoid profiling and MD simulations, we have elucidated a high-confidence interactome that links PTPN1 inhibition to the disruption of ER homeostasis and tumor suppression. Future studies should focus on the in vivo validation of this axis and the potential for combining DHE with existing immunotherapies.

## 4. Materials and Methods

### 4.1. Cell Lines and Cell Culture

Human bladder cancer cell lines T24 and 5637 were utilized in this study. Cells were obtained from the American Type Culture Collection (ATCC, Manassas, VA, USA), including the T24 cell line (ATCC Cat. No. HTB-4) and the 5637 cell line (ATCC Cat. No. HTB-9), and were cultured in RPMI-1640 medium (Gibco, Thermo Fisher Scientific, Waltham, MA, USA) supplemented with 10% fetal bovine serum (FBS; Sigma-Aldrich, St. Louis, MO, USA) and 1% penicillin-streptomycin (100 U/mL; Gibco, Thermo Fisher Scientific, Waltham, MA, USA). Cells were maintained at 37 °C in a humidified incubator with 5% CO_2_.

### 4.2. Chemical Compounds and Probe Synthesis

DHE was purchased from Targetmol (Shanghai, China). DHE is a 2,3-dihydro-1-benzofuran-type neolignan bearing two stereogenic centers at C-2 and C-3. Previous studies have reported this scaffold both as racemic trans-dehydrodiisoeugenol [(±)-licarin A] and as resolved enantiomeric forms [16,32,33]. The commercial DHE used in this study was not supplied with stereochemical assignment or chiral purity data, and no independent chiral characterization was performed in the present work. Therefore, no definitive absolute configuration was assigned to the experimental material. Synthesis of DHE-Probe: A photoaffinity probe was synthesized to retain the biological activity of the parent compound while enabling target capture. The synthesis involved incorporating a diazirine group (photo-cross-linker) and a terminal alkyne handle (click chemistry tag) onto the DHE scaffold. The coupling reaction was performed using a diazirine–alkyne linker(3-(But-3-yn-1-yl)-3-(2-iodoethyl)-3H-diazirine, Shanghai Macklin Biochemical Technology Co., Ltd., Shanghai, China) and K_2_CO_3_ in DMF at 65 °C for 16 h, yielding the final DHE-Probe with a yield of 43.7%. The chemical structure and synthetic route are detailed in Figure 3B.

### 4.3. Cell Viability and Proliferation Assays

Cell viability was assessed using the Cell Counting Kit-8 (CCK-8) assay (P-CA-001, Procell). T24 and 5637 cells were seeded in 96-well plates at a density of 5 × 10^3^ cells per well and allowed to adhere for 24 h. Subsequently, cells were treated with indicated concentrations of DHE or vehicle control (0.1% DMSO, *v*/*v*) for 48 h. To avoid interference from residual drugs, cells were washed once with phosphate-buffered saline (PBS) before the addition of 100 μL medium containing 10% CCK-8 reagent. After incubation at 37 °C for 2 h, the absorbance was measured at 450 nm using a Synergy H1 multimode microplate reader (BioTek Instruments, Winooski, VT, USA). The final concentration of DMSO in all experimental groups was strictly maintained at 0.1% to eliminate solvent-induced cytotoxicity. For proliferation kinetics, cells were treated similarly, and viability was measured every 24 h for 5 consecutive days.

### 4.4. Colony Formation Assay

Cells were seeded at low density in 6-well plates and treated with DMSO or 40 μM DHE. After incubation to allow colony formation, cells were fixed, stained, and the number and size of colonies were quantified.

### 4.5. Wound Healing Migration Assay

Cell migration was evaluated using a wound-healing assay. T24 and 5637 cells were grown to confluence, and a scratch wound was created using a pipette tip. Cells were washed to remove debris and treated with DHE (0, 10, 20, 40 μM). Images were captured at 0 h and 24 h. The wound closure area was quantified using ImageJ 1.53c to calculate the percentage of area recovery.

### 4.6. Patient-Derived Organoid Culture

3D bladder cancer organoids were established from patient tumor tissues (Ethics approval: 2024041710245802) as previously described. Briefly, organoids were suspended in growth-factor-reduced Matrigel and seeded into 96-well white-walled opaque plates. Organoids were treated with DMSO or DHE (20, 40 μM) for 72 h. Growth inhibition and viability were quantified using a luminescence-based ATP detection kit (Yeasen Biotechnology Co., Ltd., Shanghai, China) according to the manufacturer’s instructions. Briefly, 100 μL of reagent was added to each well, followed by 10 min of incubation to induce cell lysis and stabilize the luminescent signal. Relative Light Units (RLU) were measured using a microplate reader. Morphological changes were observed and captured via bright-field microscopy at 0 and 72 h post-treatment.

### 4.7. Activity-Based Protein Profiling (ABPP)

T24 cell lysates were incubated with the DHE-Probe (0–80 μM) to assess concentration-dependent labeling. Samples were then irradiated with UV light at 365 nm to induce covalent cross-linking between the diazirine moiety of the probe and interacting proteins. Subsequently, the labeled proteome was conjugated with either biotin-azide or fluorescent-azide via Copper(I)-catalyzed Azide-Alkyne Cycloaddition (CuAAC). Biotinylated proteins were enriched using streptavidin beads. The enriched proteins were analyzed via SDS-PAGE followed by Coomassie Brilliant Blue (CBB) staining or Western blotting, or subjected to LC-MS/MS for protein identification. For target validation, lysates were pre-incubated with an excess of unlabeled DHE prior to the addition of the DHE-Probe to assess competitive binding and verify target specificity. For ABPP-based mass spectrometry analysis, streptavidin-enriched proteins from the DHE-Probe, DMSO control, and DHE competition groups were processed in parallel. Briefly, the enriched proteins were subjected to reduction and alkylation, followed by tryptic digestion and peptide desalting. The resulting peptides were analyzed via LC-MS/MS using a high-resolution mass spectrometry platform. Raw data were searched against the UniProt human database using a standard proteomics data analysis workflow, and peptide and protein identifications were filtered at a false discovery rate (FDR) of ≤1%. Proteins enriched in the DHE-Probe group relative to the DMSO control and reduced in the DHE competition group were considered candidate DHE-binding proteins.

### 4.8. Global Proteomics Analysis via DIA LC-MS/MS

Global proteomic profiling was performed on DHE-treated bladder cancer organoids with three biological replicates per group (*n* = 3). Proteins were extracted using a lysis buffer containing 8 M urea and 1% SDS supplemented with protease inhibitors. After trypsin digestion and peptide desalting, the samples were analyzed using an Orbitrap Astral mass spectrometer (Thermo Fisher Scientific, Waltham, MA, USA) coupled with a Vanquish Neo LC system. Peptides were separated on a uPAC High Throughput column (75 µm × 5.5 cm, Thermo) and acquired in Data-Independent Acquisition (DIA) mode with a scan range of 100–1700 *m*/*z*.

The raw DIA data were processed using DIA-NN (v1.8). Protein identification was performed with a False Discovery Rate (FDR) of ≤1% at both peptide and protein levels. To gain biological insights, the identified proteins and their sequences were annotated against seven major databases: EggNOG, Gene Ontology (GO), Kyoto Encyclopedia of Genes and Genomes (KEGG), NR, Pfam, String (v11.5), and UniProt, as well as specialized subcellular localization databases. Protein quantification was achieved using the MaxLFQ method. For statistical analysis, protein intensities were Log2-transformed. Significantly differentially expressed proteins (DEPs) were defined using a student’s *t*-test with thresholds of *p* < 0.05 and a fold change (FC) >1.2 or <0.83. Subsequently, enrichment analysis for GO and KEGG terms was performed based on a hypergeometric distribution using Fisher’s Exact Test. The background dataset consisted of all proteins identified in the organoid samples. To control for false discovery, *p*-values were adjusted for multiple testing using the Benjamini-Hochberg (BH) method, with a significance threshold of FDR < 0.05.

### 4.9. Cellular Thermal Shift Assay (CETSA) and Western Blot

Streptavidin-enriched proteins from DHE-Probe-treated lysates (with or without DHE competition) were analyzed via Western blotting using antibodies against PTPN1. Cellular Thermal Shift Assay (CETSA): T24 cell lysates treated with DMSO or DHE were subjected to a temperature gradient (40 °C to 72 °C). The soluble fractions were analyzed by Western blotting to assess the thermal stabilization of PTPN1, indicating direct drug binding.

### 4.10. Molecular Docking and Molecular Dynamics (MD) Simulations

To address the stereochemical inconsistency identified during revision, four stereoisomers of DHE, namely, trans-(2R,3R)-DHE, cis-(2R,3S)-DHE, cis-(2S,3R)-DHE, and trans-(2S,3S)-DHE, were constructed and individually evaluated in the in silico analysis. Each stereoisomer was docked into PTPN1, and the resulting protein–ligand complexes were subjected to 100 ns molecular dynamics (MD) simulations using GROMACS 2024.4. The PTPN1 protein topology was generated using the AMBER14SB force field. The ligand topologies were generated using sobtop 1.0 (dev3.1) with the GAFF2 force field, and partial charges were assigned using the RESP method. Comparative analyses included Root Mean Square Deviation (RMSD), Root Mean Square Fluctuation (RMSF), Radius of Gyration (Rg), Solvent Accessible Surface Area (SASA), intermolecular hydrogen bonds, and free energy landscape (FEL) analysis. Because the commercial DHE used in the biological experiments was not supplied with stereochemical assignment or chiral purity data, this computational comparison was used to evaluate plausible binding modes rather than to assign the absolute stereochemistry of the experimental material.

### 4.11. Statistical Analysis

All quantitative data are presented as Mean ± Standard Deviation (SD) derived from at least three independent biological replicates (*n* = 3). Statistical significance was determined using Student’s *t*-test for two-group comparisons or one-way ANOVA followed by Tukey’s post hoc test for multiple-group comparisons. *p*< 0.05 was considered statistically significant.

## Data Availability

The proteomics data generated in this study have been deposited in the China National Center for Bioinformation (CNCB) under project accession number PRJCA059391.

## Supplementary Materials

The following supporting information can be downloaded at: https://www.mdpi.com/article/10.3390/ph19040651/s1. Figure S1: Intermolecular hydrogen-bond numbers, Figure S2: Free energy landscape (FEL) analysis.
