# Supplementary material for: Identification and Functional Analysis of Targets of Dehydrodiisoeugenol in Bladder Cancer Based on Chemoproteomics-Based Profiling
Source: Pharmaceuticals (Basel). 2026 Apr 21;19(4):651. doi: 10.3390/ph19040651 (PMC13118370; doi:10.3390/ph19040651)
Supplement: Supplementary file 1 [file pharmaceuticals-19-00651-s001.zip › pharmaceuticals-4171248-supplementary.pdf]

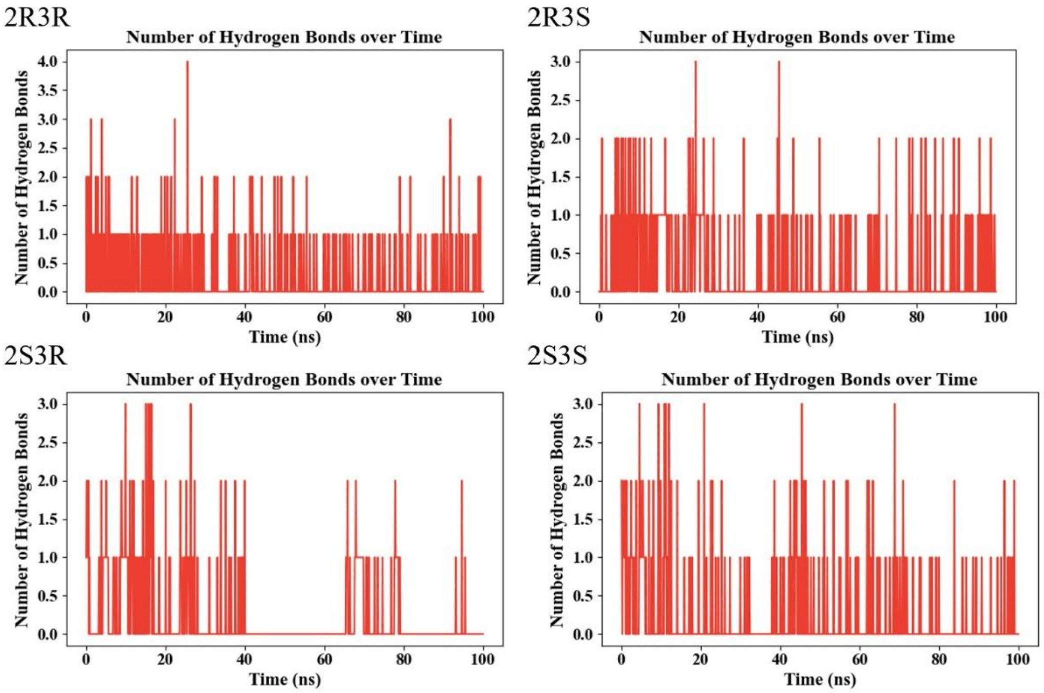

Supplementary Figure S1. Intermolecular hydrogen-bond numbers.

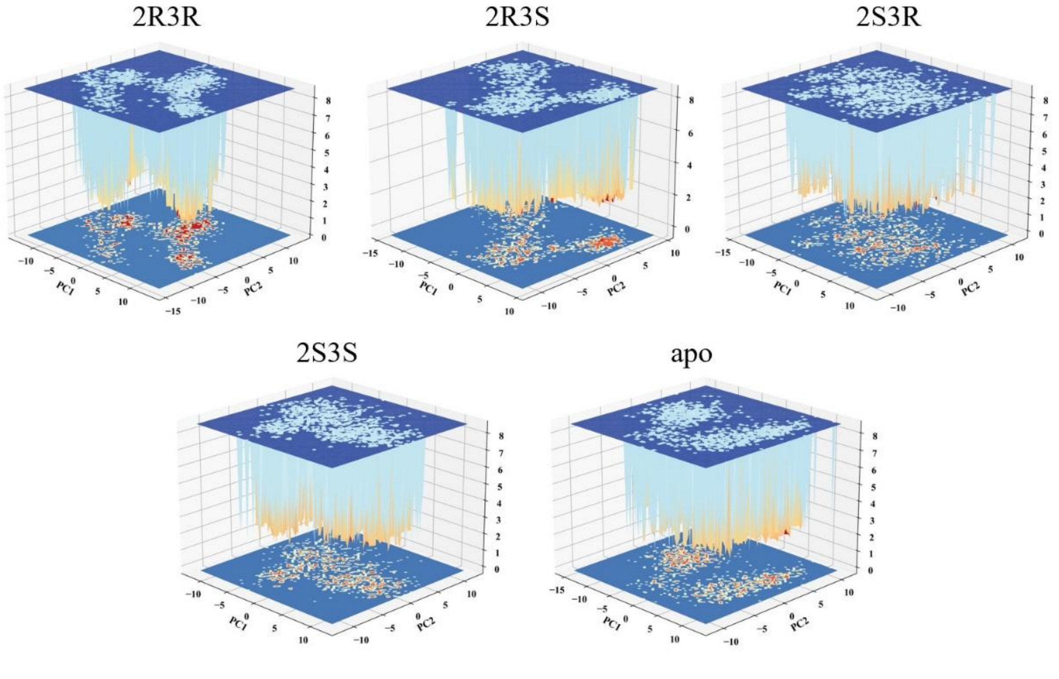

Supplementary Figure S2. Free energy landscape (FEL) analysis.
